# ¿Qué proponen los médicos para mejorar la coordinación entre niveles? Resultados en seis países de Latinoamérica

**DOI:** 10.15446/rsap.V26n1.106956

**Published:** 2024-01-01

**Authors:** Aida Oliver-Anglés, Ingrid Vargas-Lorenzo, Julieta López-Vázquez, Isabella Chagas-Samico, Daniela Campaz-Landazábal, Amparo S. Mogollón-Pérez, Pamela Eguiguren-Bravo, Delia I. Amarilla, Fernando Bertolotto, María Luisa Vázquez

**Affiliations:** 1 AO: Lic. Ciencias Políticas. M. Sc. Salud Pública. Consorci de Salut i Social de Catalunya. Barcelona, España. aoliver@consorci.org Consorci de Salut i Social de Catalunya Consorci de Salut i Social de Catalunya Barcelona España aoliver@consorci.org; 2 IV: Lic. Economía. M.Sc. Salud Pública. Ph. D. Salud Pública. Consorci de Salut i Social de Catalunya. Barcelona, España. ivargas@consorci.org Consorci de Salut i Social de Catalunya Salud Pública Consorci de Salut i Social de Catalunya Barcelona España ivargas@consorci.org; 3 JL: OD. M. Sc. Salud Pública. Instituto de Salud Pública, Universidad Veracruzana. Veracruz, México. julieta_lv_uv@hotmail.com Universidad Veracruzana Instituto de Salud Pública Universidad Veracruzana Veracruz México; 4 IC: MD. Pediatra. M. Sc. Salud Materno Infantil. Grupo de Estudio de Gestión y Evaluación de la Salud, Instituto de Medicina Integral Profesor Fernando Figueira. Recife, Brasil. isabella@imip.org.br Instituto de Medicina Integral Profesor Fernando Figueira Grupo de Estudio de Gestión y Evaluación de la Salud Instituto de Medicina Integral Profesor Fernando Figueira Recife Brasil isabella@imip.org.br; 5 DC: MD. M. Sc. Salud Pública. Consorci de Salut i Social de Catalunya. Barcelona, España. ydcampaz@consorci.org Consorci de Salut i Social de Catalunya Salud Pública Consorci de Salut i Social de Catalunya Barcelona España ydcampaz@consorci.org; 6 AM: FT. Adm. Empresas. Ph. D. Salud Pública e Investigación Biomédica. Escuela de Medicina y Ciencias de la Salud, Universidad del Rosario. Bogotá, Colombia. amparo.mogollon@urosario.edu.co Universidad del Rosario Escuela de Medicina y Ciencias de la Salud Universidad del Rosario Bogotá Colombia amparo.mogollon@urosario.edu.co; 7 PE: Matrona. M. Sc. Salud Pública y Gestión Sanitaria. Ph. D. Salud Pública. Escuela de Salud Pública Dr. Salvador Allende Gossens, Facultad de Medicina, Universidad de Chile. Santiago de Chile, Chile. peguiguren@u.uchile.cl Universidad de Chile Facultad de Medicina Universidad de Chile Santiago de Chile Chile peguiguren@u.uchile.cl; 8 DA: Antrop. Social. M. Sc. Salud Pública. Centro de Estudios Interdisciplinarios, Universidad Nacional de Rosario. Rosario, Argentina delia.inesam@gmail.com Universidad Nacional de Rosario Centro de Estudios Interdisciplinarios Universidad Nacional de Rosario Rosario Argentina; 9 FB: Lic. Enfermería. Facultad de Enfermería, Universidad de la República. Montevideo, Uruguay. fbertolotto@gmail.com Universidad de la República Facultad de Enfermería Universidad de la República Montevideo Uruguay; 10 MV: MD. Esp. Medicina Preventiva y Salud Pública. M. Sc. Políticas de Salud. Ph.D. Medicina y Cirugía Educación. Consorci de Salut i Social de Catalunya. Barcelona, España. mlvazquez@consorci.org Consorci de Salut i Social de Catalunya Medicina y Cirugía Educación Consorci de Salut i Social de Catalunya Barcelona España mlvazquez@consorci.org

**Keywords:** Investigación sobre servicios de salud, América Latina, atención primaria de salud, atención secundaria de salud (fuente: DeCS, BIREME), Health services research, Latin America, primary health care, secondary care *(source: MeSH, NLM)*

## Abstract

**Objetivo:**

Analizar las estrategias para la mejora de la coordinación clínica propuestas por médicos/as de atención primaria (AP) y especializada (AE) de redes públicas de servicios de salud de seis países de América Latina, y sus cambios entre el 2015 y 2017.

**Métodos:**

Se analizaron dos estudios transversales basados en encuestas (2015 y 2017) con aplicación del cuestionario COORDENA a un total de 4 311 médicos/as de atención primaria y especializada de dos redes públicas de servicios de salud de cada país. Se realizó un análisis descriptivo estratificado por país y año de las sugerencias propuestas.

**Resultados:**

En todos los países se señala la introducción o mejora de estrategias y mecanismos que facilitan la interacción y el conocimiento mutuo entre profesionales de distintos niveles, especialmente las reuniones conjuntas internivel, los mecanismos de comunicación directa y estrategias para fortalecer el uso de la hoja de referencia y contrarreferencia. Con menor frecuencia, se sugieren otras estrategias organizativas orientadas al fortalecimiento del modelo de atención primaria, la mejora del acceso a atención especializada y la coordinación del nivel directivo de la red.

**Conclusiones:**

Los resultados permiten generar recomendaciones para la mejora de la coordinación clínica en distintos sistemas de salud, a partir de las sugerencias de los médicos/as, una perspectiva poco tenida en cuenta en el diseño e implementación de intervenciones en los servicios de salud. Se señala la necesidad de promover estrategias basadas en la retroalimentación mutua en la elaboración de políticas públicas sanitarias.

## Abstract (en)

**Objective:**

To analyze the strategies for improving clinical coordination proposed by primary care (PC) and secondary care (SC) doctors from the public healthcare networks in six Latin American countries, and the changes between 2015 and 2017.

**Methods:**

Two cross-sectional studies based on surveys (2015 and 2017) were developed with the application of the COORDENA questionnaire to a total of 4 311 primary and secondary care doctors from two public healthcare networks in each country. A descriptive analysis of the proposed suggestions was carried out stratified by country and year.

**Results:**

In all countries, the introduction or improvement of strategies and mechanisms that ease interaction and mutual knowledge between professionals from different care levels is noted, especially joint cross-level meetings, direct communication mechanisms and strategies to strengthen the use of the referral and back-referral form. Less frequently, other organisational strategies are suggested aimed at strengthening the primary care model, access to secondary care and the coordination of the network's management.

**Conclusions:**

The results allow to generate recommendations for the improvement of clinical coordination in different health systems drawn from the suggestions made by doctors, a perspective that is seldom considered in the design and implementation of interventions in healthcare services. The need to promote strategies based on mutual adjustment in the development of public health policies is highlighted.

Las consecuencias de la fragmentación en la provisión de la atención han convertido la coordinación clínica (CC) en una prioridad para muchos sistemas de salud, particularmente en Latinoamérica 1. Con diferencias entre países, la evidencia muestra una limitada CC en la región 2, con deficiencias en el intercambio de información entre niveles de atención 3, desacuerdos en el tratamiento o la referencia 4 y dificultades de acceso a la atención especializada 5. En respuesta, se han introducido numerosas iniciativas, desde la promoción de redes integradas de salud a los mecanismos de coordinación clínica (MCC) 1. Estos últimos pueden basarse en la estandarización de habilidades (formación), procesos (guías compartidas de práctica clínica) o resultados; o en la retroalimentación, donde destacan mecanismos de adaptación mutua, como la comunicación directa (teléfono, correo electrónico), los dispositivos de enlace (comités de gestión, directivos integradores) y los sistemas de información vertical (historia clínica compartida, hoja de referencia y contrarreferencia (HRCR), epicrisis) 6, aunque su implementación es limitada en la región 7.

La CC entre niveles de atención se define como la concertación de todos los servicios relacionados con la atención en salud, con independencia del lugar donde se reciban, de manera que se armonicen y se alcance un objetivo común sin conflictos 8. Se identifican dos tipos: a) coordinación de la información clínica (transferencia y uso de la información del paciente); y b) coordinación de la gestión clínica, entendida como la provisión secuencial y complementaria de servicios a lo largo del continuo asistencial 6. La CC es un fenómeno complejo, influido por factores organizativos (p. ej., implementación de mecanismos de coordinación y disponibilidad de tiempo para usarlos) y relacionados con los profesionales, tanto individuales (demográficos, laborales) como de interacción (conocimiento y confianza en los profesionales del otro nivel) 2,9,10. Finalmente, las características del sistema de salud en el que opera la organización también desempeñan un papel relevante 11.

La implementación de MCC se ha basado tradicional-mente en enfoques de arriba abajo, convirtiendo a los profesionales en sujetos pasivos ante los cambios organizativos. Sin embargo, cada vez existe más evidencia de que la efectividad y la sostenibilidad de las intervenciones dependen de su adaptación al contexto y de la participación de los profesionales en su diseño e implementación 12,13, debido a su experiencia en la práctica clínica diaria y el conocimiento del sistema de salud. A pesar de ello, estudios recientes en Latinoamérica apuntan a que su escasa participación en el diseño de MCC podría explicar su limitado uso 7,14,15.

La aproximación poco participativa en este ámbito se refleja también en los escasos estudios que analizan estrategias propuestas por los profesionales de la salud para mejorar la CC, inexistentes en Latinoamérica. La mayoría se limita a enfermedades o mecanismos concretos 16,17 o se ha realizado en otros contextos 9,18,19. Estos estudios destacan la importancia de los MCC, especialmente los basados en la retroalimentación, y de las intervenciones que contribuyen a la mejora de los factores de interacción y organizativos.

El proyecto Equity-LA II 20 evaluó la efectividad de distintas estrategias, diseñadas e implementadas mediante un proceso de investigación-acción-participativa (IAP), en la mejora de la CC en Latinoamérica. El presente estudio, enmarcado en este proyecto y que complementa análisis previos 2,3,7,13,21,22, tiene como objetivo analizar las estrategias para la mejora de la CC propuestas por médicos/as de atención primaria (AP) y especializada (AE) de redes públicas de servicios de salud de Argentina, Brasil, Chile, Colombia, México y Uruguay, y sus cambios entre el 2015 y el 2017.

## MÉTODO

### Diseño y área de estudio

Se llevó a cabo un análisis comparativo de dos estudios transversales basados en encuestas (2015, 2017), con la aplicación del cuestionario COORDENA^®^ a médicos/as de AP y AE de Argentina, Brasil, Chile, Colombia, México y Uruguay. En cada país el área de estudio estaba compuesta por dos redes públicas de servicios de salud, seleccionadas aplicando los criterios: a) provisión de un continuo de servicios incluyendo al menos AP y AE; b) población definida; c) áreas urbanas de nivel socioeconómico bajo-medio bajo; d) voluntad de participar 20.

### Población de estudio y muestra

La población de estudio eran médicos/as de AP y AE cuya práctica diaria conllevaba el contacto con médicos/as del otro nivel y con antigüedad en la red superior a tres meses. Se estimó un tamaño muestral de 348 médicos/as por país (174/red) para asegurar la detección de diferencias del 15%, con un 80% de potencia estadística y un nivel de confianza del 95%. La selección se hizo a partir de la lista de médicos/as que trabajaba en los centros. El porcentaje que rechazó participar osciló entre el 2,6% en Colombia y el 7,6% en Uruguay en 2015; y el 3,2% en Chile y el 11,6% en México en 2017 21.

### Recogida de datos

Los datos se recogieron mediante la aplicación presencial del cuestionario COORDENA® 23, que incluye una pregunta abierta para recoger sugerencias de mejora de la CC en la red, objeto de análisis del presente artículo. La recogida de datos se realizó de mayo a octubre de 2015 y de octubre de 2017 a mayo de 2018. Los detalles sobre el diseño, la estructura, la adaptación y la validación del cuestionario ya han sido publicados 2,3.

### Análisis

Se realizó un análisis descriptivo de las respuestas a la pregunta abierta "¿Qué propone para mejorar la coordinación de la atención entre los médicos/as de atención primaria y especialistas de la red?", estratificado por país y año. Para ello, se crearon códigos mediante la revisión de una muestra de respuestas en cada país y se aplicaron para codificar el total de respuestas, creándose nuevos cuando fue necesario. Se clasificaron según áreas temáticas, a partir de las frecuencias y del marco teórico del estudio 24,25, para cada país y año. Se realizó un análisis bivariado para identificar cambios en las características de las muestras entre 2015 y 2017.

### Consideraciones éticas

El estudio fue aprobado por los comités éticos de los países participantes. Los entrevistados participaron voluntariamente tras firmar un consentimiento informado. Se garantizó el anonimato, la confidencialidad, la protección de datos y el derecho a retirarse del estudio en cualquier momento.

## RESULTADOS

Los nombres de los países se utilizan para facilitar la descripción de los resultados, y se refieren únicamente a las áreas de estudio.

### Características de la muestra

Un total de 2 160 médicos/as participó en la encuesta en 2015 (2017=2151). Destacó la mayor proporción de mujeres en Argentina (69,7%) y de hombres en Colombia (65,3%); siendo más equilibrada en el resto de países. La distribución etaria fue similar, excepto en Colombia, donde predominaron los médicos/as jóvenes (52,1%) y en México, donde lo hicieron los mayores de 50 años (51,5%). Más de la mitad en todos los países trabajaba en AE, llevaba más de tres años en la red y tenía un contrato indefinido, excepto en Chile y Colombia, con mayor proporción de médicos/as con antigüedad inferior a un año (33%) y contrato temporal (61,2% y 78%, respectivamente). El mayor porcentaje de médicos/as contratados más de 40 horas/semana se encontró en Colombia (55,9%), y el de menos de 20 horas, en Uruguay (57,5%). En relación con factores organizativos, solo una minoría declaró tener tiempo suficiente para la CC (< 40%), especialmente en Chile y Colombia (< 20%). La mayoría no conocía a los profesionales del otro nivel, excepto en Uruguay (29,5%); pero confiaba en sus habilidades clínicas (> 50%), especialmente en Argentina (76%) y Uruguay (81,9%). Alrededor de la mitad identificaba al médico de AP como responsable del seguimiento del paciente, principalmente en Argentina (72,9%).

Se identificaron algunas diferencias significativas (p < 0,05) en 2017. En Argentina, una mayor proporción de médicos/as trabajaba en AP (56,3%) y tenía menos de un año de experiencia en la red (21,6%), igual que en Brasil (29%). Se identificó mayor temporalidad en Brasil (42,5%) y menor en México (10,5%). En Chile, un mayor porcentaje de médicos/as declaró trabajar más de 40 horas/semana (53,5%). En Colombia, un mayor porcentaje declaró tener tiempo suficiente para la CC (23,3%). Los factores de interacción mejoraron principalmente en Brasil, donde declararon con mayor frecuencia conocer a los médicos/as del otro nivel, confiar en sus habilidades e identificar AP como responsable del seguimiento del paciente (Tabla 1).

**Tabla 1:** Características de la muestra de estudio

	Argentina		Brasil		Chile		Colombia		México		Uruguay	
	N=350	N=352	N=381	N=362	N=348	N=361	N=363	N=362	N=365	N=363	N=353	N=351
	n (%)	n (%)	n (%)	n (%)	n (%)	n (%)	n (%)	n (%)	n (%)	n (%)	n (%)	n (%)
Sexo												
Mujer	244(69,7)	264(75,0)	220(57,7)	207(57,2)	166(47,7)	171(47,4)	126(34,7)	123(34,0)	163(44,7)	145(39,9)	191(54,1)	205(58,4)
Hombre	106(30,3)	88(25,0)	161(42,3)	155(42,8)	182(52,3)	190(52,6)	237(65,3)	239(66,0)	202(55,3)	218(60,1)	161(45,6)	146(41,6)
Edad												
<35 años	78(22,3)	87(24,7)	104(27,3)	112(30,9)	133(38,2)	167(46,3)	189(52,1)	162(44,8)	38(10,4)	31(8,5)	60(17,0)	59 (16,8)
36-50 años	160(45,7)	174(49,4)	155(40,7)	148(40,9)	126(36,2)	112(31,0)	94(25,9)	116(32,0)	139(38,1)	119(32,8)	166(47,0)	161(45,9)
>50 años	112(32,0)	91(25,9)	118(31,0)	101(27,9)	89(25,6)	82(22,7)	78(21,5)	84(23,2)	188(51,5)	213(58,7)	121(34,3)	131(37,3)
Nivel de atención												
Atención primaria	157(44,9)	198(56,3)	109(28,6)	124(34,3)	141(40,5)	166(46,0)	118(32,5)	126(34,8)	156(42,7)	148(40,8)	109(30,9)	116(33,0)
Atención especializada	193(55,1)	154(43,8)	272(71,4)	237(65,5)	207(59,5)	195(54,0)	245(67,5)	236(65,2)	209(57,3)	215(59,2)	244(69,1)	235(67,0)
Tiempo trabajando en el centro												
1 año o menos	47(13,4)	76(21,6)	73(19,2)	105(29,0)	117(33,6)	98(27,1)	120(33,1)	120(33,1)	27(7,4)	32(8,8)	35(9,9)	26(7,4)
De 1 a 3 años	61(17,4)	67(19,0)	112(29,4)	71(19,6)	61(17,5)	82(22,7)	98(27,0)	78(21,5)	40(11,0)	33(9,1)	54(15,3)	46(13,1)
Más de 3 años	242(69,1)	207(58,8)	196(51,4)	186(51,4)	170(48,9)	181(50,1)	145(39,9)	164(45,3)	298(81,6)	298(82,1)	264(74,8)	279(79,5)
Tipo de contrato												
Indefinido	267(76,3)	246(69,9)	292(76,6)	208(57,5)	129(37,1)	112(31,0)	73(20,1)	81(22,4)	295(80,8)	324(89,3)	258(73,1)	254(72,4)
Temporal	80(22,9)	101(28,7)	89(23,4)	154(42,5)	213(61,2)	246(68,1)	283(78,0)	281(77,6)	69(18,9)	38(10,5)	83(23,5)	96(27,4)
Horas contratadas/semana												
20 o menos	54(15,4)	61(17,3)	165(43,3)	166(45,9)	34(9,8)	24 (6,6)	41(11,3)	22(6,1)	2(0,5)	1(0,3)	203(57,5)	180(51,3)
De 20 a 40 horas	279(79,7)	266(75,6)	187(49,1)	186(51,4)	173(49,7)	144(39,9)	119(32,8)	124(34,3)	351(96,2)	354(97,5)	118(33,4)	127(36,2)
Más de 40	17(4,9)	25(7,1)	29(7,6)	10(2,8)	141(40,5)	193(53,5)	203(55,9)	216(59,7)	12(3,3)	8(2,2)	32(9,1)	39(11,1)
Tiempo para coordinacióna	107(30,6)	103(29,3)	139(36,5)	156(43,1)	49(14,1)	51(14,1)	61(16,8)	84(23,3)	95(26,0)	115(31,7)	137(38,8)	132(37,6)
Conoce a médicos del otro nivel^a^	114(32,6)	114(32,4)	42(11,0)	65(18,0)	33(9,5)	30(8,3)	26(7,2)	51(14,1)	42(11,5)	64(17,6)	249(70,5)	236(67,2)
Confía médico del otro nivela	266(76,0)	286(81,3)	197(51,7)	223(61,6)	182(52,3)	218(60,4)	204(56,2)	228(63,0)	182(49,9)	196(54,0)	289(81,9)	284(80,9)
AP responsable de seguimiento^a^	255(72,9)	281(79,8)	202(53,0)	235(64,9)	196(56,3)	225(62,3)	189(52,1)	184(50,8)	195 53,4)	210(57,9)	186(52,7)	206(58,7)

^a^ Se muestran los resultados del Sí (siempre+muchas veces). En negrita: diferencias con respecto a 2017 p<0,05.

### Sugerencias de mejora de la coordinación clínica

Las propuestas para la mejora de la CC más frecuentes en 2015 fueron comunes en todos los países y se referían a factores y MCC que facilitan la retroalimentación entre profesionales de distintos niveles (Tabla 2).

**Tabla 2:** Sugerencias de mejora de la coordinación clínica entre niveles de atención^1^

	Argentina		Brasil		Chile		Colombia		México		Uruguay	
	2015	2017	2015	2017	2015	2017	2015	2017	2015	2017	2015	2017
	N=542	N=446	N=545	N=551	N=794	N=862	N=582	N=481	N=628	N=573	N=433	N=342
	n (%)	n (%)	n (%)	n (%)	n (%)	n (%)	n (%)	n (%)	n (%)	n (%)	n (%)	n (%)
Reuniones conjuntas entre profesionales de distintos niveles	138(25,5)	138(30,9)	79(14,5)	131(23,8)	107(13,5)	151(17,5)	118(20,3)	157(32,6)	104(16,6)	84(14,7)	99(22,9)	54(15,8)
Estrategias organizativas para mejorar el trabajo en red	101(18,6)	24(5,4)	49(9,0)	57(10,3)	50(6,3)	65(7,5)	56(9,6)	32(6,7)	72(11,5)	44(7,7)	28(6,5)	42(12,3)
Fomentar la comunicación y conocimiento entre médicos de distintos niveles	91(16,8)	103(23,1)	76(13,9)	81(14,7)	192(24,2)	218(25,3)	77(13,2)	40(8,3)	151(24,0)	130(22,7)	88(20,3)	60(17,5)
Mejorar la HRCR y otros mecanismos de intercambio de información clínica	85(15,7)	88(19,7)	153(28,1)	130(23,6)	144(18,1)	135(15,7)	130(22,3)	64(13,3)	91(14,5)	61(10,6)	101(23,3)	82(24,0)
Mejorar el acceso de los pacientes. entre niveles y factores asociados	61(11,3)	44(9,9)	44(8,1)	45(8,2)	66(8,3)	51(5,9)	21(3,6)	19(4,0)	37(5,9)	34(5,9)	48(11,1)	43(12,6)
Mejorar la infraestructura para la transferencia de información clínica	24(4,4)	27(6,1)	66(12,1)	61(11,1)	80(10,1)	98(11,4)	65(11,2)	91(18,9)	43(6,8)	79(13,8)	47(10,9)	38(11,1)
Realizar sesiones formativas.	24(4,4)	6(1,3)	50(9,2)	37(6,7)	98(12,3)	81(9,4)	69(11,9)	53(11,0)	112(17,8)	119(20,8)	1(0,2)	5(1,5)
Implementar/mejorar mecanismos de coordinación de la gestión clínica	18(3,3)	16(3,6)	28(5,1)	9(1,6)	44(5,5)	53(6,1)	39(6,7)	24(5,0)	3(0,5)	16(2,8)	17(3,9)	8(2,3)
9. Otras sugerencias de mejora2	-	-	-	-	13(1,6)	10(1,2)	7(1,2)	1(0,2)	15(2,4)	6(1,0)	4(0,9)	10(2,9)

^1^ La tabla muestra la distribución de las respuestas por categoría, ordenadas según la frecuencia obtenida en Argentina, 2015. La pregunta admitía un número ilimitado de respuestas por informante. ^2^La categoría *otras sugerencias de mejora* agrupa las categorías de respuestas con frecuencias inferiores al 1,5%.

Concretamente, se referían a la *organización de reuniones conjuntas,* especialmente en Argentina (25,5% del total de las sugerencias), Uruguay (22,9%) y Colombia (20,3%); el *fomento de la comunicación* y del conocimiento mutuo, sobre todo en Chile (24,2%), México (24%) y Uruguay (20,3%); la mejora de la HRCR y otros mecanismos de intercambio de información clínica, especialmente en Brasil (28,1%), Uruguay (23,3%) y Colombia (22,3%); y, con menor frecuencia, la mejora de la infraestructura para la transferencia de información, especialmente en Brasil (12,1%). Se sugirió también la realización de sesiones formativas, principalmente en México (17,8%), y otras estrategias organizativas para mejorar el trabajo en red, especialmente en Argentina (18,6%). En menor medida, los profesionales destacaron la importancia de mejorar el acceso de los pacientes entre niveles en Argentina (11,3%) y Uruguay, (11,1%) y de implementar y mejorar los MCC de la gestión clínica (p. ej., protocolos, guías de práctica clínica) en Colombia (6,7%), Chile (5,5%) y Brasil (5,1%).

En 2017 aumentaron las propuestas sobre reuniones conjuntas en todos los países, excepto en México y Uruguay, de fomento de la comunicación y del conocimiento, especialmente en Argentina, y la mejora de la infraestructura para la transferencia de la información, excepto en Brasil. Las dos primeras se convirtieron en las sugerencias más frecuentes en las redes participantes, excepto en Uruguay, donde aumentó la mejora de la HRCR y otros mecanismos de intercambio de información (Tabla 2).

Estas propuestas se refieren a tres ámbitos -comunicación y conocimiento mutuo, mejora del uso de la HRCR y otras estrategias organizativas- que, por su frecuencia, se han podido analizar en mayor detalle (Tabla 3).

**Tabla 3:** Sugerencias por ámbito temático^1^

	Argentina		Brasil		Chile		Colombia		México		Uruguay	
	2015	2017	2015	2017	2015	2017	2015	2017	2015	2017	2015	2017
	n (%)	n (%)	n (%)	n (%)	n (%)	n (%)	n (%)	n (%)	n (%)	n (%)	n (%)	n (%)
(3,6) Fomentar la comunicación y conocimiento mutuo entre profesionales	N=115	N=130	N=142	N=142	N=272	N=316	N=142	N=131	N=194	N=209	N=135	N=98
Mejorar la actitud, predisposición a coordinarse y conocimiento mutuo	16(13,9)	18(13,8)	3(2,1)	3(2,1)	71(26,1)	66(20,9)	2(1,4)	1(0,8)	67(34,5)	39(18,7)	19(14,1)	16(16,3)
Mejorar la comunicación y medios para ello	75(65,2)	85(65,4)	71(50,0)	77(54,2)	82(30,1)	77(24,4)	75(52,8)	37(28,2)	84(43,3)	81(38,8)	69(51,1)	44(44,9)
Fomentar la expansión/uso de mecanismos de comunicación directa	24(20,9)	27(20,8)	52(36,6)	53(37,3)	119(43,8)	173(54,7)	59(41,5)	84(64,1)	13(6,7)	66(31,6)	27(20,0)	21(21,4)
Disponibilidad de teléfono, PC y/o internet	-	-	-	-	-	-	-	-	11(5,7)	8(3,8)	20(14,8)	17(17,3)
Directorio de contactos de profesionales de la red	-	-	16(11,3)	9(6,3)	-	-	6(4,2)	9(6,9)	19(9,8)	15(7,2)	-	-
(4) Mejorar la HRCR y otros mecanismos de intercambio de información clínica	N=85	N=88	N=153	N=130	N=144	N=135	N=130	N=64	N=91	N=61	N=101	N=82
Uso de la HRCR y epicrisis	50(58,5)	53(60,2)	60(39,2)	39(30,0)	20(13,9)	17(12,6)	-	-	-	2(3,3)	54(53,5)	29(35,4)
Desarrollar estrategias organizativas para mejorar el uso de la HRCR	5(4,9)	1(1,1)	54(35,3)	71(54,6)	57(39,6)	46(34,1)	55(42,3)	30(46,9)	86(95,0)	53(86,9)	21(20,8)	17(20,7)
Mejorar el contenido de la HRCR	-	-	17(11,1)	19(14,6)	9(6,3)	25(18,5)	16(12,3)	5(7,8)	-	-	6(5,9)	9(11,0)
Informatizar la historia clínica compartida	30(29,1)	34(38,6)	22(14,4)	1(0,8)	58(40,3)	47(34,8)	59(45,4)	29(45,3)	5(5,0)	6(9,8)	20(19,8)	27(32,9)
(2,5) Otras estrategias organizativas para mejorar el trabajo en red y el acceso	N=162	N=68	N=93	N=102	N=116	N=116	N=77	N=51	N=109	N=78	N=76	N=85
Coordinación de las funciones directivas de la red	32(19,8)	17(25,0)	2(2,2)	-	22(19,0)	19(16,4)	16(20,8)	17(33,3)	28(25,7)	20(25,6)	12(15,8)	10(11,8)
Mejorar las condiciones laborales de los médicos	1(0,6)	-	3(3,2)	6(5,9)	9(7,8)	17(14,7)	10(13,0)	9(17,6)	10(9,2)	4(5,1)	5(6,6)	4(4,7)
Reforzar el modelo de atención basado en AP	68(42,0)	7(10,3)	44(47,3)	51(50,0)	19(16,4)	29(25,0)	30(39,0)	6(11,8)	34(31,2)	19(24,4)	11(14,5)	28(32,9)
Mejorar el acceso a la AE	43(26,5)	40(58,8)	18(19,4)	16(15,7)	7(6,0)	11(9,5)	8(10,4)	6(11,8)	4(3,7)	5(6,4)	18(23,7)	24(28,2)
Aumentar los recursos humanos y materiales	17(10,5)	4(5,9)	22(23,7)	27(26,5)	39(33,6)	28(24,1)	9(11,7)	8(15,7)	25(22,9)	24(30,8)	24(31,6)	19(22,4)
Implementar mecanismos de coordinación del acceso	1(0,6)	-	4(4,3)	2(2,0)	20(17,2)	12(10,3)	4(5,2)	5(9,8)	8(7,3)	6(7,7)	6(7,9)	-

^1^ La tabla muestra la distribución de respuestas por ámbito temático, sobre el total de respuestas de la/s categorías que los conforman (numeración entre paréntesis).

#### a) Fomento de la comunicación y el conocimiento mutuo entre los profesionales de la red

La propuesta más frecuente en casi todos los países en 2015 fue la mejora de la comunicación entre profesionales. En el caso de Chile, la mayor frecuencia correspondió al fomento y uso de mecanismos de comunicación directa (p. ej., el teléfono y el correo electrónico institucionales o las pasantías en el otro nivel) (43,8%). En algunos países también se mencionó la disponibilidad de teléfono, PC e internet o de un directorio de contactos de los profesionales de la red. Otras propuestas incluyeron factores de interacción, como mejorar la actitud, la predisposición a coordinarse y el conocimiento mutuo, especialmente en Chile y México (26,1% y 34,5%, respectivamente).

El cambio más relevante en 2017 fue el aumento del fomento en la expansión y en el uso de mecanismos de comunicación directa en Chile (54,7%), Colombia (64,1%) y México (31,6%). Dentro de esta categoría, en Chile destacaron la telemedicina y las pasantías en AP y AE.

#### b) Mejora en el uso de la HRCR y otros mecanismos de intercambio de la información clínica

Algunos países hicieron hincapié en la importancia de utilizar HRCR y epicrisis (Argentina, 58,5%; Uruguay, 53,5%; Brasil, 39,2%). Principalmente en México (95%), Colombia (42,3%) y Chile (39,6%), destacaron las estrategias organizativas para mejorar el uso de la HRCR (obligatoriedad y concienciación de los profesionales sobre su uso, capacitaciones para mejorar la derivación, entre otros) y la mejora de su contenido, especialmente en Colombia (12,3%). La informatización de la historia clínica compartida fue otra de las propuestas frecuentes en Colombia (45,4%) y Chile (40,3%).

En 2017 aumentaron las estrategias organizativas en Brasil (54,6%), la informatización de la historia clínica en Argentina (38,6%) y Uruguay (32,9%) y la mejora del contenido de la HRCR en Chile (18,5%).

#### c) Desarrollo de otras estrategias organizativas para mejorar el trabajo en red y el acceso a los servicios

En 2015 emergieron diversidad de estrategias relativas a la organización de la atención y de los recursos en la red, la coordinación del acceso entre niveles y la coordinación de las funciones de dirección. Con relación al primer ámbito, la más frecuente en todos los países fue reforzar el modelo de atención basado en AP, especialmente en Argentina (42%), Brasil (47,3%) y Colombia (39%). Para ello, se propuso definir con claridad el rol y la cartera de servicios de cada nivel, reforzar el papel de la AP en el seguimiento del paciente, la asignación de especialistas de referencia a la AP en Brasil, o mejorar la integración de algunos servicios concretos. También en este ámbito destacó el aumento de los recursos humanos y materiales, particularmente en Chile (33,6%) y Uruguay (31,6%), y la mejora de las condiciones laborales de los profesionales en Colombia (13%). Se propuso mejorar el acceso de los pacientes a la AE, principalmente en Argentina (26,5%) y Uruguay (23,7%) y, especialmente en Chile, imple-mentar mecanismos de coordinación del acceso (17,2%). Finalmente, en casi todos los países se sugirió la mejora de la coordinación de las funciones directivas de la red, especialmente en México (25,7%) y Colombia (20,8%).

En 2017, esta última estrategia destacó en Colombia (33,3%), mientras que el aumento de los recursos lo hacía en México (30,8%), la mejora del acceso a la AE en Argentina (58,8%) y el fortalecimiento del modelo de atención basada en AP en Uruguay (32,9%).

## DISCUSIÓN

La fragmentación asistencial es uno de los principales problemas que enfrenta la provisión de servicios de salud en Latinoamérica, con consecuencias para la equidad en el acceso y la eficiencia 25. La evidencia muestra una limitada CC en la región 2,11 y señala la necesidad de reforzar la implantación de MCC, creando las condiciones adecuadas para utilizarlos y buscando estrategias que mejoren su efectividad 7,26. Por su rol protagonista en la CC, la participación de los médicos/as en la selección, en el diseño y en la implementación es esencial para la introducción de intervenciones que respondan a su contexto. Según nuestro conocimiento, este estudio es el primero en analizar las estrategias para la mejora de la CC propuestas por médicos/as de AP y AE en Latinoamérica, y uno de los pocos a nivel internacional 18.

Los resultados señalan la importancia de la interacción entre profesionales de distintos niveles para mejorar la CC. Aunque con diferencias según el contexto, sugirieron como estrategias principales el fomento de la comunicación directa y el conocimiento mutuo, y la implementación o mejora de MCC que contribuyan a ello, como las reuniones conjuntas, los mecanismos de comunicación directa y estrategias para fortalecer el uso de la HRCR. Destacaron también estrategias organizativas para mejorar el modelo de atención basado en AP y la coordinación del acceso en la red.

### Estrategias basadas en la retroalimentación mutua para mejorar la coordinación clínica

Las estrategias propuestas basadas en la retroalimentación coinciden con las recomendadas por la teoría de las organizaciones 27 para situaciones con actividades especializadas y alto flujo de información, frecuentes en la atención a los pacientes 24. En estas situaciones, resulta más adecuado coordinarse mediante comunicación directa, para resolver los problemas donde se genera la información, en contraposición con la coordinación basada en la estandarización, adecuada en situaciones de baja incertidumbre 24. El conocimiento mutuo entre profesionales, limitado en la mayoría de las redes analizadas 2, también ha sido destacado por diversas disciplinas 28 como un aspecto clave para reforzar el trabajo conjunto, porque ayuda a compartir experiencias, contextos y habilidades 29. El análisis de los determinantes de la coordinación en las redes de estudio 2,3 y otros contextos 10,18 muestra que una mejor comunicación se asocia a una mejor percepción de la coordinación y al uso frecuente de MCC, junto con factores de interacción como la confianza en las habilidades de los profesionales del otro nivel o identificar al médico de AP como responsable del paciente, también sugeridas por los profesionales.

El mecanismo propuesto con más frecuencia fue la organización de reuniones conjuntas internivel, prácticamente inexistente en las redes de estudio en 2015 7. Esta fue también la estrategia seleccionada posteriormente por los profesionales que participaron en el proceso IAP en el marco del Equity-LA II. En cinco de las redes se imple-mentaron reuniones conjuntas en diferentes modalidades para enfrentar los problemas de coordinación identificados: falta de comunicación, limitado intercambio de información y desacuerdos en la gestión clínica 22.

Otra estrategia sugerida con frecuencia fue mejorar la comunicación directa entre niveles mediante el uso del teléfono, el correo electrónico y la disponibilidad del directorio de contactos de la red, también escasamente implementados en las redes de estudio, excepto en Argentina y Uruguay 7. Estos mecanismos son importantes para asegurar la coordinación de la información cuando se requiere una respuesta rápida 13, y puede traducirse en una mejora de la consistencia de la atención, el seguimiento de los pacientes y la resolutividad 30.

La evaluación de las intervenciones en las redes de estudio 13 mostró, por un lado, que las reuniones conjuntas fomentaron el uso de mecanismos de comunicación directa entre los asistentes. Por otro, en línea con otros estudios 19, las intervenciones basadas en la retroalimentación no solo favorecían la coordinación de la información y gestión clínica. Además, mejoraban factores de interacción como el conocimiento mutuo y la confianza entre médicos/as. Esto explicaría el aumento en 2017, en casi todos los países, de la sugerencia de realizar más reuniones conjuntas y promover los mecanismos de comunicación directa.

### Estrategias de mejora del uso de la HRCR para reforzar la transferencia de información entre niveles

Los resultados señalan la necesidad de implementar estrategias para mejorar el uso de la HRCR, principal mecanismo para el intercambio de información 7. Aunque un análisis previo 3 identificó un elevado conocimiento de este instrumento por los médicos/as en casi todas las redes de estudio, también señaló deficiencias en su uso, especialmente en la contrarreferencia desde AE. Estas deficiencias tienen importantes consecuencias para la calidad de la atención 31. En Brasil y Uruguay el nivel de conocimiento del mecanismo fue inferior 7, lo que explicaría la elevada frecuencia de sugerencias relacionadas en ambos contextos, y que este mecanismo fuese el foco de la intervención participativa en Uruguay 22. La evidencia 31 recomienda la introducción combinada de muchas de las estrategias propuestas para mejorar el uso de la HRCR -sesiones formativas, concienciación de su relevancia, mejora del contenido y disponibilidad del formato-, recalcando los factores que determinan su uso, particularmente la interacción entre los médicos/as de diferentes niveles 3.

### Retroalimentación entre las estrategias de fortalecimiento del modelo de atención basado en AP y la coordinación clínica

La coordinación de la información y de la gestión clínica es clave para el funcionamiento adecuado del modelo de atención basado en AP 32. A su vez, la adhesión al modelo por parte de los médicos/as de AP y AE influye en la predisposición a colaborar con el otro nivel 2,3. Por tanto, implementar estrategias que mejoren la CC fortalece el modelo y viceversa. Los profesionales han señalado estrategias de mejora en esta línea, como la definición de los roles de cada nivel (p. ej., a través de capacitaciones o de espacios de retroalimentación), o el apoyo de la AE en el rol de la AP como coordinadora de la atención (p. ej., a través de la asignación de especialistas de referencia). Sin embargo, también reconocen la importancia para fortalecer el seguimiento del paciente por parte de AP de la disminución de los tiempos de espera a la AE, y la mejora de la estabilidad laboral en países con elevada rotación de profesionales y condiciones laborales precarias 2,33. Esto último favorece otros aspectos clave del modelo, como la continuidad de relación con los pacientes y los profesionales de los otros niveles 34.

## Limitaciones

El tamaño muestral del estudio impidió realizar los análisis por nivel de atención, lo que habría resultado de interés dada la distinta experiencia de la CC entre médicos/as de AP y AE. No obstante, la evidencia reciente no identificó diferencias significativas entre las sugerencias propuestas por los profesionales de ambos niveles 18. Asimismo, el tamaño de la muestra impidió analizar la significación estadística de las diferencias entre las sugerencias propuestas por país y año.

A pesar de sus limitaciones, este estudio contribuye a generar conocimiento sobre estrategias de mejora de la CC a partir de las opiniones de sus usuarios principales: los médicos/as, cuya perspectiva no suele considerarse en el diseño de estas estrategias. En línea con la evidencia, los resultados muestran la importancia para los profesionales del fortalecimiento de la comunicación directa, el conocimiento mutuo y la implementación de MCC basados en la retroalimentación. Entre ellos, destacan las reuniones conjuntas, los mecanismos de comunicación directa, y estrategias para fortalecer el uso de la HRCR, coincidiendo con las estrategias diseñadas e implementadas posteriormente de forma participativa. Los cambios entre años apuntan a los beneficios de las intervenciones percibidos por los médico/as y la conveniencia de extenderlos a toda la red para mejorar la CC. Estos resultados son relevantes para la elaboración de políticas públicas en el ámbito sanitario y para las redes públicas de servicios de salud ♠
